# Repulsion between Oppositely Charged Planar Macroions

**DOI:** 10.1371/journal.pone.0069436

**Published:** 2013-08-05

**Authors:** YongSeok Jho, Frank L. H. Brown, MahnWon Kim, Philip A. Pincus

**Affiliations:** 1 Asia Pacific Center for Theoretical Physics, Pohang, Gyeongbuk-do, Korea; 2 Department of Physics, Pohang University of Science and Technology, Pohang, Gyeongbuk-do, South Korea; 3 Department of Physics, University of California Santa Barbara, Santa Barbara, California, United States of America; 4 Department of Chemistry and Biochemistry, University of California Santa Barbara, Santa Barbara, California, United States of America; 5 Department of Physics, Korea Advanced Institute of Science and Technology, Yuseong-Gu, Daejeon, Korea; 6 Materials Research Laboratory, University of California Santa Barbara, Santa Barbara, California, United States of America; University of Zurich, Switzerland

## Abstract

The repulsive interaction between oppositely charged macroions is investigated using Grand Canonical Monte Carlo simulations of an unrestricted primitive model, including the effect of inhomogeneous surface charge and its density, the depth of surface charge, the cation size, and the dielectric permittivity of solvent and macroions, and their contrast. The origin of the repulsion is a combination of osmotic pressure and ionic screening resulting from excess salt between the macroions. The excess charge over-reduces the electrostatic attraction between macroions and raises the entropic repulsion. The magnitude of the repulsion increases when the dielectric constant of the solvent is lowered (below that of water) and/or the surface charge density is increased, in good agreement with experiment. Smaller size of surface charge and the cation, their discreteness and mobility are other factors that enhance the repulsion and charge inversion phenomenons.

## Introduction

In solution, charged macroions are expected to be surrounded by a cloud of compensating oppositely charged counterions [1], [2]. Within the context of mean-field theory (Poisson-Boltzmann theory [3] or Debye-Huckel theory [4]) this cloud acts somewhat intuitively to reduce the effective net charge of the macroion monotonically with distance from the macroion surface. At large distances from the macroion surface, the electrostatic field generated by the macroion is completely screened by the counterions, corresponding to a vanishing net charge associated with the mean-field macroion/counterion complex. As a corollary to this behavior, like charged macroions will always repel one another and unlike charged macroions will always attract one another within mean-field theory (albeit with reduced force from the unscreened case). It is well known, however, that the mean-field description is inadequate in the context of highly charged surfaces and multivalent counterions are often encountered in biological systems [5]–[10]. Indeed, the mean-field description is qualitatively incorrect; attraction between like-charged macroions [11] and inversion of macroion polarity [12], [13] are both observable under certain conditions.

Recently, it is found that the highly charged macroions can be over-screened by multivalent ions leading to the excessive compensation of the surrounding charge cloud [5], [14], [15]. It is analogous to the strong aggregation between highly charged biocomplexes, for example nucleosomes [5] or layer by layer polyelectrolyte adsorption. A direct measurement of repulsion between two oppositely charged macroions was carried out by Besteman *et al.* [12], [13] using force microscopy. They observed repulsion between a negatively charged mica surface and a positively charged amine-terminated surface, when the concentration of trivalent (or higher valence) salts exceeded a critical concentration. It was further established that this critical ion concentration is reduced by decreasing the dielectric constant of the solvent surrounding the macroions and by increasing the surface charge density of the macroions. These authors interpreted their results within the context of a one component plasma model (OCP) developed by Nguyen and Shklovskii [16], [17] and suggested the origin of the repulsion as a charge inversion on the silica surface (the cations were multivalent in the experimental studies, whereas the anions were monovalent).

Since these experiments appeared in the literature, there has been some additional computational/theoretical studies of the charge inversion by the multivalent cations or salts. There were studies to understand the charge renormalization in the frame of the DH theory [18]–[22]. Allahyarov *et al.* [23] showed the coexisting phase, condensation and redissolution of DNA bundles in presence of the tetravalent cations and monovalent salt. Dahirel and Hanse [24] first found the repulsive force for triplets when one of the polyions has opposite charge. They showed the possibility of the instability in condition that multivalent ions are near the oppositely charge polyions.

More direct comparison with the Besteman's experiments has been made by Trulsson *et al.* [25]. They demonstrated the possibility of repulsion between two oppositely charged macroions in the context of numerical simulations, but noted that the phenomenon does not need to be directly correlated with charge inversion. They extended their previous work considering secondary monovalent salts, and changing the dielectric permittivity. It is noted that at a distance where the repulsion starts, the net charge is not inverted. The charge inversion comes at a longer distance [26]. And, Hatlo and Lue developed a field theoretic model [27], which also supports the possibility of repulsion of opposite charges.

In this paper, we extend previous studies by including the effect of discreteness of surface charges (as opposed to a uniform surface charge density), and their mobility, the size of cation and the surface charge, and the dielectric permittivity of solvent and macroions and their contrast. It has been known that the effect of the discreteness of surface charge and the dielectric permittivity of the solvent and macroion and their contrast contribute the system significantly. Meyer and Delville [28] found that there is a strong internal correlation in the condensed layers of divalent cations when the surface charge density is not uniform. Taboada-Serrano *et al.* [29], [30], Calero and Faraudo [31] revealed that the discreteness modifies the electrostatics, for example, attraction between like-charged colloids is enhanced by the discreteness and the asymmetry of the surface charge distribution. Wu *et al.* [32] reported that the potential of mean force between oppositely charged colloids becomes stronger in presence of multivalent cations. Li and Wu [33] showed that the charge inversion is caused by an adsorption of polyelectrolytes using nonlocal density functional theory. The dielectric discontinuity modifies the interaction significantly. Linse [34] showed that the colloid in higher dielectric medium undergoes a repulsion due to the multipoles generated by the dielectric discontinuities.

Ion size is also important in the charge screening and the colloidal interaction. Ravindran and Wu [35] showed that smaller divalent cations enhance the attraction between like-charged colloids, especially the short ranged attraction. Martin-Molina *et al.* [36] also found the strong dependence of the ion size on the interaction by measuring the mean force between charged plates. They found the transition of the interaction between like-charged plates from attraction to repulsion as increasing ion size. Not only the bulk ion size, but also the depth of the surface charges gives strong impact on the interaction and the charge inversion [9], [31].

We improve upon the numerical methods by considering a fully periodic simulation geometry in the directions parallel to the macroion interface while explicitly considering the dielectric contrast between water and the macromolecules (as in reference [37]). In our previous article, we contrasted that our potential calculation method which counts the dielectric discontinuity gives significant difference to the Ewald or similar type of method which doesn't consider the dielectric discontinuity [37]. It is found that the dielectric contrast with lower dielectric permitivity of solvent, and the discreteness of surface charge and its depth can increase the charge inversion and the repulsion.

We find that discrete surface charge enhances the repulsion between macroions and we are able to reproduce the experimental trends relating solvent dielectric contrast to the critical concentration of multivalent ions necessary to observe repulsion. The smaller cation or shallower depth of surface charge brings more cations to the oppose surface, and enhances the repulsion. However, if the cations are too small, the anions can recombine with the cation to reduce the cation's effective valence. This effect leads to diminished repulsion for very small cations.

This paper is organized as follows: In next section, we describe the model system and the numerical methods employed in the simulation. Then, we present numerical results for pressure vs. distance curves and directly compare our predictions for the critical of ions necessary to induce repulsion to experiment. Lastly, we discuss our results and conclude.

## Results

### Description of the simulation model

**Figure 1:**
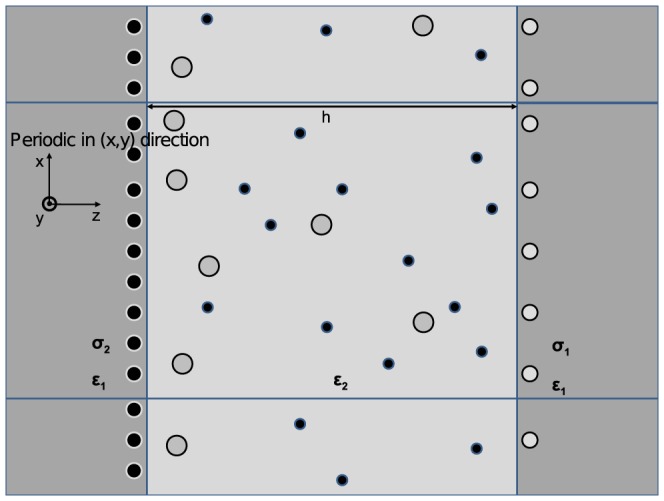
Schematic for the model system. Two oppositely charged macroions are separated by a distance 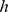. The surface charge densities for the two macroions are 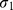 and 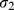, where 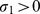 and 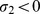 and 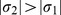. The displayed image considers the case where 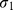 and 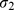 result from the placement of mobile discrete monovalent surface charges (white and black circles). For comparison, we also consider the case of a homogeneous surface charge density where all charges are smeared into a continuum, see text. The interstitial region between the two macroions is occupied by mobile 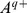 particles (large grey circles) to guarantee neutrality of the simulation box as well as additional mobile 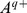 and 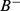 particles (small black circles) present in the stoichiometry of the 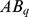 salt. In addition to Coulombic forces, hard core repulsions are present between all ion pairs and between the ions and the macroion surfaces. The macroions and solvent are treated as a uniform dielectric materials with dielectric constants of 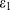 and 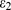, respectively. The simulation box is periodic in the 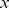 and 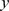 directions parallel to the macroion surfaces. For the purposes of electrostatic calculations, the 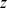 dimension is assumed to extend to 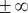 (i.e. infinitely thick macroions), however all mobile particles are confined to the narrow 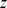 band reflecting the solvent region.

The model system is similar to those described in Fig. 1. The system is composed of two oppositely charged planar macroions with faces oriented parallel to one another, separated by a distance 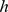. The negatively charged surface has higher charge density than the positively charged surface, leading to a net negative charge associated with the two macroion surfaces. 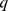-valent cation, 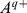 , between the two macroions balance total system charge. Charge neutrality must be obeyed and the 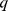-valent cations compensate the net surface charges exactly. Additional salt molecules, 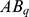 , may also be present in the interstitial region as governed by the chemical potential of the salt (see below). These molecules are assumed to be completely dissociated into the component ions (i.e. 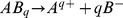), but are always present in the stoichiometry of the salt to preserve neutrality of the simulation box. Unless noted otherwise, the surface charge densities of macroions are taken as 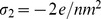 and 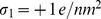 [25]. Both uniform and discrete surface charge models are considered. In the case of the discrete model, the monovalent surface charges were arranged 2.5*Å* beneath the solvent/macroion interface; in the case of the uniform charge density, the charge is localized to the macroion/solvent interface. Both the bulk solvent and the macroions are treated as uniform dielectric materials, whose dielectric constants are different. Temperature is set at 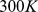 and the corresponding Bjerrum length, 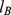, (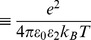) when the dielectric constant is 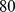 (the default value we use for pure aqueous solution) is about 7*Å*. The ions present in solution, 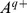 and 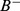, are modeled as hard spheres with radii chosen as 4*Å* and 2*Å* respectively. The size of the simulation is determined by the surface charge density and the number of cations which neutralize the system. We present the number of cations at later. The total number of salts are changed by the Widom's rule. 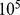 steps are used for equilibration and another 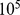 steps are used for the data production. Other details are presented in the text and figure caption, when it is appeared.

Fully simulating typical macroions, for example the silica sphere used in refs. [12], [13] whose diameter is of order of ten microns, is prohibitive computational expense. Instead, we introduce the planar geometry discussed above and assume periodic boundary conditions in the 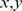 directions parallel to the surface orientation. As all mobile particles are restricted to the region between the macroions, it is unnecessary to introduce periodic conditions in the 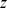 direction. The necessary electrostatic calculations are handled in a manner discussed previously [9], [37], which allows for numerically exact calculations within the geometry described, even when the dielectric constants for the solution and macroions differ from one another.

### Discreteness of surface charge

**Figure 2:**
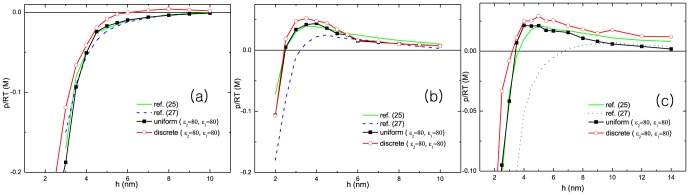
The pressure between two planar macroions are plotted as a function of distance under the different conditions of surface charge distribution. Simulation results are compared with those of references [25], [27]. 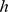 is a distance between two macroions defined in Fig. 1. The surface charge densities are 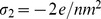 and 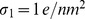. The radii of cation and anion are 4*Å* and 2*Å* each. The cation valence varies 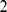 in (a), 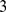 in (b), and 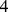 in (c). Salt concentrations are 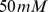 for (a), 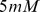 for (b), and 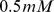 for (c). The dielectric constants are uniform over the simulation space and chosen as 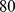.

In Fig. 2, the pressure between two planar macroions is plotted as a function of their separation. The pressure is measured by the following equation:
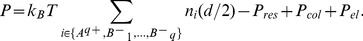
(1)The first term describes the entropic/osmotic pressure due to the salt concentration at the midplane. 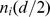 are the concentration of species 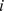 at the midplane. 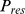 is the pressure at reservoir which is obtained from the separated simulations in bulk under constant salt concentration using the virial theorem. 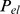 is the total electrostatic pressure between charges in left half and charges in right half. It contains not only free charges, but also charges on surfaces. 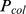 is the hard core collision pressure [38]. A rigorous derivation of this formula using pressure tensor method is presented at the reference [39]. 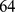 multivalent cations are considered in the bulk in addition to the salt particles. The corresponding periodic length of the simulation box in lateral directions is 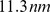 for divalent salt, 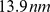 for trivalent salt, and 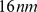 for tetravalent salt. The number of surface charges changes according to the cation valence, for example, when 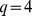, 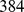 negative charges for 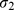 and 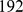 positive charges for 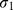 are present. The number of salt particle varies according to the salt concentration keeping the chemical potential constant.

First of all, we compare our simulations with the previous theoretical [27] and numerical [25] results which assume the surface charges are uniform. We observed the repulsion between two oppositely charged macroions. The origin of the repulsion is the combined effect of the reduction in electrostatic interaction by excess charges and their entropic repulsion. Our result qualitatively agrees with previous numerical study [25] and the theoretical result [27] under the same conditions. We also found when the repulsion starts the net charge is still anionic. The charge inversion is observed at larger distance which also agrees with previous studies. Here, the charge inversion distance is defined as a distance 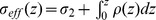 turns to positive. 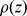 is the net charge density at 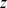.

In Fig. 2, we consider the discreteness of surface charge. Each figure displays results of combination of uniform, discrete and mobile surface charge distribution. It is known that the discreteness of surface charge density enhances the strong electrostatic interaction between macroions and cations. In case of two like-charged macroions, the attraction between two macroions is promoted by the discreteness [9] of surface charges, *ie.* the charge correlation effect is enhanced. In addition, the mobility of surface charges also increases the charge correlation. The additional degrees of freedom in surface charge brings more salt particles to the surfaces. In the following subsections, more details will be seen to depend on these factors.

### Charged particle size

**Figure 3:**
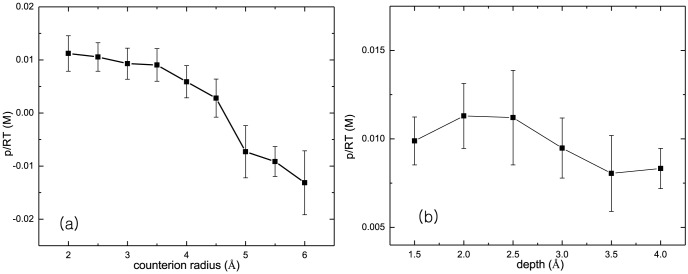
The pressure versus (a) cation size, and (b) depth of surface charge are presented. The surface charge densities are 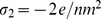 and 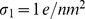. The radii of cation and anion are 4*Å* and 2*Å* each. The cation valence varies 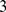, and the salt concentrations are 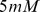. The separation between surfaces is kept as 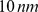. The dielectric constants are chosen as 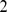 for macroion and 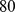 for solvent.

Fig. 3 shows the pressure distance curves depending on the cation size, and the depth of surface charge. As presented in File S1, the condensation of multivalent cations is function of their radius and the depth of surface charge. When the charged particles are small, either cation or surface charge, stronger binding of cation to the charged surface is eligible which leads consequent overcondensation of cations onto surface. However, if the cation size is too small, then the anions are partially recombined with cations to reduce the effective cation valence. Indeed, the overall chemical potential does not vary much. This explains the slow change in pressure with respect to the cation size at 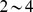 *Å*, in Fig. 3. Increasing the cation size more, the strong coupling effect reduces and when it is over 5*Å*, the pressure eventually turns to be attractive.

In the Fig. 3-b), the depth of surface charge is varying while the cation size is kept constant, 4*Å*. The smaller depth of surface charge also reduces the chemical potential of cations as in File S1. However, because 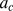 is larger than 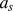, the change in surface charge depth does not contribute on the chemical potential much, only by sub-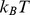. Thus, the results show that the pressure is a bit higher at shallower depth, but the change is not large.

### Dielectric inhomogeneity between solvent and the macromolecules

We next compare the pressures obtained from the system where the dielectric constant between macroions and solvent is inhomogeneous with those obtained from the system where it is homogeneous. There exists an intrinsic strong dielectric contrasts between high dielectric constant of water and the low dielectric constant of macroions. It has shown that the electrostatic interaction is modified by the dielectric contrast [40], [41], and especially it becomes important near highly charged surfaces [37]. Besteman [12], [13] experimentally found that the charge inversion and the repulsion strongly depend on the dielectric constant of solvent.

**Figure 4:**
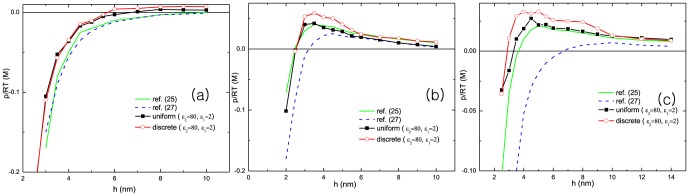
The pressure between two planar macroions are plotted as a function of distance under the different conditions of dielectric contrast. Simulation results are compared with those of references [25], [27]. The surface charge densities are 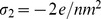 and 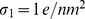. The radii of cation and anion are 4*Å* and 2*Å* each. The cation valence varies 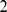 in (a), 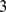 in (b), and 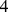 in (c). Salt concentrations are 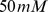 for (a), 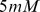 for (b), and 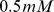 for (c). The dielectric constants are chosen as 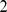 for macroion and 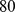 for solvent [46].

Fig. 4 shows significant differences in pressures between homogeneous and inhomogeneous dielectric distribution. The repulsion comes up at shorter distance for inhomogeneous dielectric distribution compared to the homogeneous dielectric distribution, although the peaks are established at similar distances. The electrostatic interaction from the lower dielectric surface is about twice stronger compared to the homogeneous dielectric distribution. For this reason, the multivalent cations are bound to the surface tighter. The chemical potential at surface is decreased and more cations are brought to the surface although the salt concentrations are the same. The other source of repulsion is induced surface charges (image charges). Due to the lower dielectric material of the macroions, same sign image charges are induced at the surface which makes the repulsion stronger. But this contribution decays quickly at long distance over several 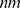. It is noted that under the consideration of surface charge discreteness the magnitude of repulsion is higher. The coupling of the dielectric contrast and the spatial inhomogeneity amplifies the fluctuation mediated correlation.

### Critical concentration

**Figure 5:**
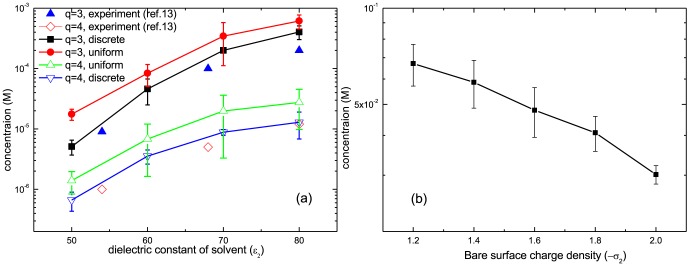
The critical concentrations of ions necessary to induce repulsion are plotted in a log scale. (a) The charge densities are 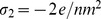 and 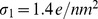. The radii of cations and anions are 4*Å* and 2*Å* each. Experimental results from reference of Besteman *et al.* [13] are presented as a comparison. The dielectric constant of the macroions is fixed as 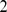. The dielectric constant of solvent varies from 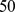 to 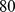 according to the experimental scheme. The cation valence changes 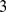 and 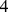 as well. Two types of surface charge distributions are considered: uniform/discrete and mobile. (b) The critical salt concentration is drawn as a function of 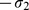 when 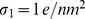. Cation is trivalent. The dielectric constant of solvent is 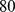 while that of macroions remains as 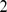.

Next, we measure the critical concentration as a function of the dielectric constant of solvent (Fig. 5-a)). Four combinations of trivalent/tetravalent cations and uniform, discrete and mobile surface charge distributions are studied. Numerical results give quantitative agreement with experimental ones, when 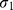 is 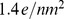. As decreasing the dielectric constant of solvent, 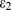, we find that the repulsion starts at lower salt concentration. The dielectric constant is a measure how strongly the solvent screens the electric field. Thus the charge interaction is stronger at lower dielectric solvent, which leads stronger coupling effects between surface charges and multivalent cations. As a result, it decreases potential near the surface, and brings more cations to the surface in order to make balance in the chemical potential. In other words, at the same salt concentration the local salt excess at surface is higher for lower dielectric solvent. In the same context, it is obvious that the critical concentration is lower in presence of tetravalent cations than trivalent cations. The electrostatic coupling is higher for tetravalent cations. We also compare the critical salt concentration for uniform surface charge with the discrete surface charge. The critical concentration significantly decreases when the surface charge is discrete. Comparing to the uniform charge surface, the discrete charge surface contributes to extra strength of attraction which enhances the local salt excess [9]. The detail of their difference is described in File S1.

In Fig. 5-b) the critical concentration is plotted versus the surface charge density. We assume that solvent is water whose dielectric constant is 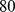, and the dielectric constant of macroions is 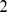. Because chemical potential is proportional to 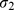, we have higher the local salt excess as increasing the surface charge density. As a result, the critical concentration drops quickly. For example, when the bare surface charge density is about 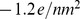, we need 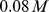 of salt concentration to have repulsion, but this drops to 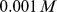 when bare surface charge density is increased to 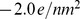. The strong dependence on the dielectric constant and the surface charge density stems from their linear or inverse linear dependence on chemical potential which has exponential dependence on the critical concentration.

In Besteman's paper, they argued how the strong correlation provokes the repulsion between two surfaces. In File S1 we extended their arguments considering the discreteness of surface charge and the dielectric contrast between water and the substrate. We find our results are qualitatively explained with these extended model.

## Discussion

The repulsion between oppositely charged macroions is studied in consideration of the inhomogeneities in surface charge distribution and the dielectric constant. In the mean field frame work, salts reduce the attraction between the macroions, but sign of the interaction does not change. Beyond mean field regime, for example, when the bare surface charge density is high (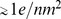) and the salts are multivalent, as increasing the salt concentration, eventually interaction turns to be repulsive. Performing intensive numerical investigation, we find that the inhomogeneities of the surface charge distribution and dielectric constant, the size of the ions are main ingredients controlling the critical concentration, and the strength of the repulsion.

The repulsion is increased enhancing the correlation of charge fluctuation, and local salt excess at surfaces. Accordingly, the critical concentration is set at lower value. Under the explicit consideration of the dielectric constant, we find that the repulsion is enhanced by lower dielectric constant of solvent which screens the electrostatic correlation less. The repulsion is also promoted by the increase of the surface charge density, and cation valence. The surface flexibility also induces stronger condensation of cations to the surface [42]. It allows the surface charges to be adjusted to the distribution of the multivalent salts in order to reduce the free energy of the system. It leads stronger repulsion of the surfaces.

Opposite charge repulsion has a similar physical background with the like charge attraction (LCA), but there exist some meaningful differences. First of all, the typical theories and conventional simulations predict the equilibrium of LCA is established at a very short distance around few Angstroms without considering dielectric contrast. It is extended to a nano meter with consideration of dielectric contrast [43]. Besides, the repulsion between oppositely charged macroions is observed in several nanometers or larger distance. Second, the attraction between like-charged surfaces turns to repulsion at large distance (at distance much longer than Bjerrum length). At larger separation the surfaces are screened by cations and the surface charge densities are renormalized to 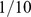 or even less [44]. It follows mean field approximation which projects repulsion only. Besides, the opposite charge repulsion is sustained to the very long distance (at least in our simulation range). At large distance, a net charge of one highly charged surface is inverted, and effectively they are looked like two like-charged macroions. Third, we need at least critical amount of salt to observe the opposite charge repulsion, contrary to the like charge attraction which is observed even in absence of the salt.

Common natural biomaterials don't have surface charge densities larger than 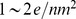. In practical condition, the opposite charge repulsion is provoked by the multiple valent salts. For example, if polyelectrolytes, charged vesicle, carry out the role of the salts, it will easily cause the excess aggregation on the surface of charged macroions, and repulsion and/or the charge inversion under the surface charge density less than 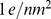 in natural system, for example, layer-by-layer polyelectrolyte assembly which spotlights for the industrial purpose [45].

## Supporting Information

File S1 **Supporting information.**
(PDF)

Figure S1 **The excess chemical potential of the *AB_q_* salt.** Numerical results are compared to the theoretical results based on the Debye-Huckel theory and its extension (see text). a) The valence of cation is fixed at q  =  3. Excess chemical potentials are plotted as a function of salt concentration. The inset is the magnification of the figure at a low concentration. b) Excess chemical potential is plotted as a function of cation valence *q*. The salt concentration is fixed at 1*mM*.
(EPS)

Figure S2 **The distributions of bulk ions are displayed.** a) The cation density is plotted from the surface 2. Cation valence is 4, and the concentration is 0.0005*M*. The charge densities are σ2  =  −2 *e/nm2* and σ1  =  1 *e/nm2*. Distance between membrane is 5°*A.* b) The net charged density from the surface 2 is plotted.
(EPS)

## References

[1] Safran S (2003) Statistical thermodynamics of surfaces, interfaces, and membranes. Westview Pr

[2] Poon W, Andelman D (2006) Soft condensed matter physics in molecular and cell biology. Taylor & Francis Group

[3] Gouy G (1910) Constitution of the electric charge at the surface of an electrolyte. J phys 9: 457–467.

[4] Debye P, Huckel E (1923) Zur theorie der elektrolyte. Phys Z 24: 185–206.

[5] Grosberg A, Nguyen T, Shklovskii B (2002) Colloquium: The physics of charge inversion in chemical and biological systems. Reviews of modern physics 74: 329–345.

[6] Moreira A, Netz R (2002) Simulations of counterions at charged plates. The European Physical Journal E: Soft Matter and Biological Physics 8: 33–58. 10.1140/epje/i2001-10091-9 15010981

[7] Ha B (2001) Modes of counterion density fluctuations and counterion-mediated attractions between like-charged fluid membranes. Physical Review E 64: 31507. 10.1103/PhysRevE.64.031507 11580342

[8] Lau A, Pincus P (2002) Counterion condensation and fluctuation-induced attraction. Physical Review E 66: 41501. 10.1103/PhysRevE.66.041501 12443206

[9] Jho Y, Park G, Chang C, Pincus P, Kim M (2006) Interaction between two inhomogeneously charged parallel surfaces in the strong coupling regime. Physical Review E 73: 21502. 10.1103/PhysRevE.73.021502 16605337

[10] Kanduč M, Trulsson M, Naji A, Burak Y, Forsman J, et al (2008) Weak-and strong-coupling electrostatic interactions between asymmetrically charged planar surfaces. Physical Review E 78: 61105. 10.1103/PhysRevE.78.061105 19256800

[11] Bloomfield V (1991) Condensation of DNA by multivalent cations: considerations on mechanism. Biopolymers 31: 1471–1481. 1814499 10.1002/bip.360311305

[12] Besteman K, Zevenbergen M, Heering H, Lemay S (2004) Direct observation of charge inversion by multivalent ions as a universal electrostatic phenomenon. Physical review letters 93: 170802. 15525062 10.1103/PhysRevLett.93.170802

[13] Besteman K, Zevenbergen M, Lemay S (2005) Charge inversion by multivalent ions: Dependence on dielectric constant and surface-charge density. Physical Review E 72: 61501. 10.1103/PhysRevE.72.061501 16485949

[14] Bungenberg de Jong H, Kruyt H (1949) Colloid science. Kruyt, H, Ed 335–432.

[15] Kabanov A, Kabanov V (1995) Dna complexes with polycations for the delivery of genetic material into cells. Bioconjugate chemistry 6: 7–20. 7711106 10.1021/bc00031a002

[16] Nguyen T, Grosberg A, Shklovskii B (2000) Screening of a charged particle by multivalent counterions in salty water: Strong charge inversion. The Journal of Chemical Physics 113: 1110.

[17] Nguyen T, Shklovskii B (2001) Complexation of DNA with positive spheres: phase diagram of charge inversion and reentrant condensation. The Journal of Chemical Physics 115: 7298.

[18] Ulander J, Greberg H, Kjellander R (2001) Primary and secondary effective charges for electrical double layer systems with asymmetric electrolytes. The Journal of Chemical Physics 115: 7144.

[19] Ramirez R, Kjellander R (2003) Dressed molecule theory for liquids and solutions: An exact charge renormalization formalism for molecules with arbitrary charge distributions. The Journal of Chemical Physics 119: 11380.

[20] Kjellander R, Mitchell D (1994) Dressed-ion theory for electrolyte solutions: A Debye–Huckel-like reformulation of the exact theory for the primitive model. The Journal of Chemical Physics 101: 603.

[21] Kjellander R (2007) Fundamental aspects of electrostatic interactions and charge renormalization in electrolyte systems. Colloid Journal 69: 20–28.

[22] Attard P (1993) Asymptotic analysis of primitive model electrolytes and the electrical double layer. Physical Review E 48: 3604–3621. 10.1103/physreve.48.3604 9961018

[23] Allahyarov E, Gompper G, Lowen H (2005) DNA condensation and redissolution: interaction between overcharged DNA molecules. Journal of Physics: Condensed Matter 17: S1827.

[24] Dahirel V, Hansen J (2009) Ion-mediated interactions in suspensions of oppositely charged nanoparticles. The Journal of chemical physics 131: 084902. 19725632 10.1063/1.3193556

[25] Trulsson M, Jonsson B, Åkesson T, Forsman J, Labbez C (2006) Repulsion between Oppositely Charged Surfaces in Multivalent Electrolytes. Physical review letters 97: 68302. 10.1103/PhysRevLett.97.068302 17026212

[26] Trulsson M, Jonsson B, Åkesson T, Forsman J, Labbez C (2007) Repulsion between oppositely charged macromolecules or particles. Langmuir 23: 11562–11569. 17918865 10.1021/la701222b

[27] Hatlo M, Lue L (2009) A field theory for ions near charged surfaces valid from weak to strong couplings. Soft Matter 5: 125–133.

[28] Meyer S, Delville A (2001) (N, V, T) Monte Carlo Study of the Electrostatic Forces between Charged Lamellae: Influence of Surface Charge Localization. Langmuir 17: 7433–7438.

[29] Taboada-Serrano P, Yiacoumi S, Tsouris C (2005) Behavior of mixtures of symmetric and asymmetric electrolytes near discretely charged planar surfaces: A Monte Carlo study. The Journal of chemical physics 123: 054703. 16108681 10.1063/1.1992484

[30] Taboada-Serrano P, Yiacoumi S, Tsouris C (2006) Electrostatic surface interactions in mixtures of symmetric and asymmetric electrolytes: A Monte Carlo study. The Journal of chemical physics 125: 054716. 16942250 10.1063/1.2238869

[31] Calero C, Faraudo J (2010) The interaction between electrolyte and surfaces decorated with charged groups: A molecular dynamics simulation study. The Journal of chemical physics 132: 024704. 20095691 10.1063/1.3289726

[32] Wu J, Bratko D, Blanch H, Prausnitz J (2000) Interaction between oppositely charged micelles or globular proteins. Physical Review E 62: 5273–5280. 10.1103/physreve.62.5273 11089089

[33] Li Z, Wu J (2006) Density functional theory for polyelectrolytes near oppositely charged surfaces. Physical review letters 96: 48302. 10.1103/PhysRevLett.96.048302 16486902

[34] Linse P (2008) Electrostatics in the presence of spherical dielectric discontinuities. The Journal of chemical physics 128: 214505. 18537431 10.1063/1.2908077

[35] Ravindran S, Wu J (2009) Ion size effect on colloidal forces within the primitive model. Condensed Matter Physics 8: 377–388.

[36] Martín-Molina A, Ibarra-Armenta JG, González-Tovar E, Hidalgo-Álvarez R, Quesada-Pérez M (2010) Monte carlo simulations of the electrical double layer forces in the presence of divalent electrolyte solutions: effect of the ion size. Soft Matter 7: 1441–1449.

[37] Jho Y, Kim M, Pincus P, Brown F (2008) A numerical study of the electrostatic properties of two finite-width charged dielectric slabs in water. The Journal of chemical physics 129: 134511. 19045109 10.1063/1.2970885

[38] Sjöström L, Åkesson T, Jönsson B (1993) Interaction and conformation of polyelectrolyte chains adsorbed on neutral surfaces. The Journal of chemical physics 99: 4739–4747.

[39] Heinz H, Paul W, Binder K (2005) Calculation of local pressure tensors in systems with many-body interactions. Phys Rev E 72: 066704. 10.1103/PhysRevE.72.066704 16486095

[40] Hatlo M, Curtis R, Lue L (2008) Electrostatic depletion forces between planar surfaces. The Journal of chemical physics 128: 164717. 18447489 10.1063/1.2908738

[41] Hatlo M, Lue L (2008) The role of image charges in the interactions between colloidal particles. Soft Matter 4: 1582–1596. 10.1039/b803783c 32907148

[42] Fleck C, Netz R (2005) Counterion density profiles at charged flexible membranes. Physical review letters 95: 128101. 16197114 10.1103/PhysRevLett.95.128101

[43] Jho Y, Kanduč M, Naji A, Podgornik R, Kim M, et al (2008) Strong-Coupling Electrostatics in the Presence of Dielectric Inhomogeneities. Physical review letters 101: 188101. 18999867 10.1103/PhysRevLett.101.188101

[44] Lau A, Lukatsky D, Pincus P, Safran S (2002) Charge fluctuations and counterion condensation. Physical Review E 65: 51502. 10.1103/PhysRevE.65.051502 12059559

[45] Schönhoff M (2003) Self-assembled polyelectrolyte multilayers. Current opinion in colloid & interface science 8: 86–95.

[46] Honig B, Sharp K, Yang A (1993) Macroscopic models of aqueous solutions: biological and chemical applications. The Journal of Physical Chemistry 97: 1101–1109.

