# Supplementary material for: Repulsion between Oppositely Charged Planar Macroions
Source: PLoS One. 2013 Aug 5;8(8):e69436. doi: 10.1371/journal.pone.0069436 (PMC3734153; doi:10.1371/journal.pone.0069436)
Supplement: File S1 — Supporting information. (PDF) [file pone.0069436.s001.pdf]

Repulsion between oppositely charged planar macroions

YongSeok Jho[1,2,*], Frank L. H. Brown[3,4], MahnWon Kim[5,6] Philip A. Pincus[5,6]
1 Asia Pacific Center for Theoretical Physics, Pohang, Gyeongbuk-do, Korea
2 Department of Physics, POSTECH, Pohang, Gyeongbuk-do, South Korea
3 Department of Physics, University of California at Santa Barbara, Santa Barbara, California, USA
4 Department of Chemistry and Biochemistry, University of California at Santa Barbara, Santa Barbara, California, USA
5 Department of Physics, Korea Advanced Institute of Science and Technology, Yuseong-Gu, Daejeon, Korea
6 Materials Research Laboratory, University of California at Santa Barbara, Santa Barbara, California, USA
∗ **E-mail: Corresponding ysjho@apctp.org**

Supporting Information

Chemical potential

Monte Carlo simulation is used to obtain chemical potential of the salt. Once the chemical potentials are present, all calculations are performed via Monte Carlo simulation within the Grand Canonical ensemble (GCMC) [1–4] to allow fluctuations in the number of AB_q particles present in the simulation box. The chemical potential for the salt as a function of bulk salt concentration was established by the Widom particle insertion method [5, 6] applied to periodic cubic geometries at constant volume ($V = L^3$) and temperature with the required salt concentration. The only distinction from traditional neutral species insertion method calculations [4] is that here each "particle insertion" corresponds to the simultaneous insertion of the $q + 1$ ions associated with a single AB_q salt molecule (one A^{q+} particle and q B^- particles) so that the salt's excess chemical potentia is expressed as (using the notation of [4]):

$$\mu_{ex_{AB_q}} = -k_BT \ln \int \{d\mathbf{s}_{A^{q+}} d\mathbf{s}_{B_1^-} \ldots d\mathbf{s}_{B_q^-}\}_{N+1} \langle \exp(-\beta\Delta\mathcal{U})\rangle_N . \quad (1)$$

Here $\mathbf{s} = \mathbf{r}/L$ is the scaled position of the particle within the simulation box and $\Delta\mathcal{U} = \mathcal{U}(\mathbf{s}_{N+1}) - \mathcal{U}(\mathbf{s}_N)$ is the difference in potential energy for the system with an extra salt molecule inserted (the component ions located at $\mathbf{s}_{A^{q+}_{N+1}}$ and $\mathbf{s}_{B^-_{N+1,i}}$ where $i = 1 \ldots q$) and the extra salt molecule absent. The $\langle \ldots \rangle_N$ average corresponds to the usual canonical average for the N salt molecule system and the integrals reflect the unweighted averaging associated with random placement of the ions within the box inherent to the particle insertion method.

Fig. S1 displays the results of the insertion method calculations. For divalent case, 100 positive ions and 200 negative ions are considered. For trivalent case, positive/negative ions are 81/243, for tetravalent they are 64/256, and 60/300 when $q = 5$. The size of the simulation box varies depending on the salt concentration. It is observed that the excess chemical potentials approach the limiting Debye-Huckel (DH) behavior [7] in the limit of low salt concentration and low valence of the ions, as expected. However, the experiments in question involve salt concentrations up to the millimolar range and trivalent and tetravalent positive ions, where substantial deviations from the DH theory are observed. For comparison we have also plotted the predictions of the extended DH theory suggested by Levin and Fisher [8]. Aqua *et al.* [9] showed that the Levin and Fisher's theory may not apply to the multivalent cations.

Our results are similar to previous studies [10, 11] and are included here to quantify the values of μ used in the GCMC simulations discussed below.

The cation aggregation to the surface

In Fig. S2, the cation density is plotted from the anionic surface. Both discreteness and the dielectric discontinuity are enhancement of cation condensation to the surface. In the right figure, the net charge which is defined as;

$$\rho_{net}(z) = \int_0^z \sum_i n_i(z)dz \tag{2}$$

is plotted. Because the anionic surface charge density is $-2\sigma_1$, it starts from -2 and ends at -1. Stronger inversion is seen in existence of the dielectric discontinuity and the discreteness of cations.

Modification in critical concentration due to the discreteness of surface charge

The electric field from the surface is screened by the condensed cations, and then charges apart from the surface see a regulated effective charged surface. Then, we divide the system into two regimes, cation condensed layer and the bulk region similar to the ref [12]. We assume that the cations are strongly interacting with the surface charges. Then, the chemical potential for multivalent cations $\mu_{+,s}$ at the condensed layer and $\mu_{+,b}$ at bulk are described as follows:

$$\begin{aligned} \mu_{+,s} &= \mu_{el,s} + \log N_s/N_{s,max} \\ \mu_{+,b} &= \mu_{el,b} + \log N_b/N_{b,max} \\ \mu_{el,s} &\approx -k_BT \times \\ & \left(1.65\Gamma + \frac{q^2 l_B}{a_c + a_s} + l_B \pi q \sigma_2 \left(\sqrt{a_c^2 + a_W^2} - a_c\right)\right) \end{aligned} \tag{3}$$

where, $\Gamma = \frac{1}{4k_BT\epsilon_2\epsilon_0}\sqrt{\left|\frac{e^3q^3\sigma}{\pi}\right|}$, and $\mu_{el,s}$, $\mu_{el,b}$ are the electrostatic contribution to the chemical potential at the condensed layer and bulk. N_s, N_b are the cation density at the surface and bulk. $N_{s,max}$, $N_{b,max}$ are the reference values of the density. a_c, a_s are the cation radius and surface charge radius. a_W is the Wigner radius. The first term of the equation 3 is from the electrostatic contribution to the chemical potential at uniform surface charge, and the others are corrections from considering the discreteness.

At critical concentration, the condensed cations completely compensate the surface charges, *i.e.* $N_s = \sigma_2/qe$. Then,

$$\begin{aligned} N_{b,0} &\sim \left|\frac{\sigma_2}{2a_c qe}\right| \times \\ & \exp\left(-1.65\Gamma - \frac{q^2 l_B}{a_c + a_s} + l_B\pi q\sigma\left(\sqrt{a_c^2 + a_W^2} - a_c\right)\right). \end{aligned} \tag{4}$$

Above this concentration, the excess salts with respect to the bulk concentration are supposed to be released from the surface by the entropy especially when the separation between the macroions is very long. Since the electrostatic interaction decays exponentially at large distance, the entropic pressure will be primary dominant. Therefore, the net pressure should be repulsive. This analogy agrees well with previous theoretical results which project the electrostatic interaction is decaying much faster than the entropic repulsion.

The discreteness generates additional terms in exponent. It reduces the critical concentration, because $a_W > a_c + a_s$ in highly charged surface system and $-\frac{q^2 l_B}{a_c+a_s} + l_B\pi q\sigma\left(\sqrt{a_c^2 + a_W^2} - a_c\right)$ keeps lower than zero. It is interesting to observe that the critical concentration depends not only on the discreteness of surface charge, but also on the cation valence and the size of the charged particles. Since $l_c < a_c$, we can assume that the separation between cation and surface charges are approximately $a_c + a_s$. Since,

Γ and l_B is inversely proportion to the ϵ_2, the critical concentration is decreasing for lower dielectric solvent. However, we would like to emphasize that this doesn't include the dielectric contrast effect which is important, especially when the separation is small. However, as long as we concentrate the critical concentration of charge inversion point which occurs at very large distance, it can be a reasonable approximation.

Considering the dielectric contrast between water and macroion, the electrostatic contribution to the chemical potential of eq. 4 is modified.

$$\begin{aligned}\mu_{el2,s} &\approx -k_B T \Bigg[\Gamma \sum_{j \neq i} \left(\frac{1}{|\vec{r}_i - \vec{r}_j|} + \frac{1}{|\vec{r}_i - \vec{r}_j - 2h_r|} \right) \\ &+ \frac{2\epsilon_2}{\epsilon_1 + \epsilon_2} \left\{ \frac{q^2 l_B}{a_c + a_s} + l_B \pi q \sigma \left(\sqrt{a_c^2 + a_W^2} - a_c \right) \right\} \Bigg] . \end{aligned} \tag{5}$$

where, $\vec{r}_i$, $\vec{r}_j$ are vector coordinate of particle i and j. h_r is the distance of cation j from the surface. ϵ_1 is a dielectric constant of the macromolecules, and ϵ_2 is a dielectric constant of the solute. The first two terms are from the electrostatic interactions between cations and the cation and the surface charges except the nearest ones within the Wigner radius. The third and fourth term corresponds to the correction of the electrostatic interaction between the cation and the it's nearest surface charges comparing to the smeared charged surface. The prefactor $\frac{2\epsilon_2}{\epsilon_1+\epsilon_2}$ comes from the dielectric contrast. If h_r is 0, $\mu_{el2,s} = \frac{2\epsilon_2}{\epsilon_1+\epsilon_2}\mu_{el,s}$. With nonzero h_r, $\mu_{el2,s} < \frac{2\epsilon_2}{\epsilon_1+\epsilon_2}\mu_{el,s}$. Since, the factor $\frac{2\epsilon_2}{\epsilon_1+\epsilon_2}$ is larger than 1 (for example, it is about 1.95 when the solute is water), the electrostatic contribution of the chemical potential is smaller than the case which do not consider the dielectric contrast. This correction can be even larger than the first term in eq. 3 which is originated from the lateral correlation.

References

1. Valleau J, Cohen L (1980) Primitive model electrolytes. I. Grand canonical Monte Carlo computations. The Journal of Chemical Physics 72: 5935.

2. van Megen W, Snook I (1980) The grand canonical ensemble Monte Carlo method applied to the electrical double layer. Journal of Chemical Physics 73: 4656–4662.

3. Shing K, Gubbins K (1982) The chemical potential in dense fluids and fluid mixtures via computer simulation. Molecular Physics 46: 1109–1128.

4. Frenkel D, Smit B (2001) Understanding molecular simulation. Academic Press, Inc. Orlando, FL, USA.

5. Widom B (1963) Some topics in the theory of fluids. The Journal of Chemical Physics 39: 2808.

6. Boulougouris G, Economou I, Theodorou D (1999) On the calculation of the chemical potential using the particle deletion scheme. Molecular physics 96: 905–913.

7. McQuarrie D (2000). Statistical Mechanics. Sausalito.

8. Levin Y, Fisher M (1996) Criticality in the hard-sphere ionic fluid. Physica A: Statistical and Theoretical Physics 225: 164–220.

9. Aqua J, Banerjee S, Fisher M (2005) Criticality in charge-asymmetric hard-sphere ionic fluids. Physical Review E 72: 41501.

10. Svensson B, Woodward C (1988) Widom's method for uniform and non-uniform electrolyte solutions. Molecular Physics 64: 247–259.

11. Vitalis A, Baker N, McCammon J (2004) Isim: a program for grand canonical monte carlo simulations of the ionic environment of biomolecules. Molecular Simulation 30: 45–61.

12. Nguyen T, Grosberg A, Shklovskii B (2000) Screening of a charged particle by multivalent counterions in salty water: Strong charge inversion. The Journal of Chemical Physics 113: 1110.

Supporting Information Legends

Figure S1 The excess chemical potential of the AB_q salt. Numerical results are compared to the theoretical results based on the Debye-Huckel theory and its extension (see text). a) The valence of cation is fixed at $q = 3$. Excess chemical potentials are plotted as a function of salt concentration. The inset is the magnification of the figure at a low concentration. b) Excess chemical potential is plotted as a function of cation valence q. The salt concentration is fixed at $1\,mM$.
Figure S2 The distributions of bulk ions are displayed. a) The cation density is plotted from the surface 2. Cation valence is 4, and the concentration is $0.0005M$. The charge densities are $\sigma_2 = -2\,e/nm^2$ and $\sigma_1 = 1\,e/nm^2$. Distance between membrane is $5\mathring{A}$. b) The net charged density from the surface 2 is plotted.
